# Formation of high-quality mixed silage from paper mulberry and wheat bran driven by the characteristics of the microbial community

**DOI:** 10.3389/fmicb.2024.1476067

**Published:** 2024-12-13

**Authors:** Wenbo Wang, Hua Tian, Yuwei Zhao, Yanshun Nie, Zibing Li, Junjie Gong, Wenjie Jiang, Yanjing Yin, Ramon Santos Bermudez, Wenxing He

**Affiliations:** ^1^School of Biological Science and Technology, University of Jinan, Jinan, China; ^2^Yantai Longda Breeding Co., Ltd., Yantai, China; ^3^Fengtang Ecological Agriculture Technology Research and Development (Shandong) Co., Ltd., Taian, China

**Keywords:** mixed ensiling, silage quality, microbial community, fermentation function, lignocellulose degradation

## Abstract

Paper mulberry (*Broussonetia papyrifera*) is a high-quality silage protein feed material that can help address feed shortages and support livestock development. Although some studies have investigated the relationships between microbial communities and silage quality, these relationships and the underlying community assembly processes remain complex, requiring further research to clarify them. Additionally, limited research has explored the relationship between microbial community fermentation functions and silage quality. In this study, we aimed to explore *B. papyrifera* and wheat bran mixed silage quality driven by the characteristics of the microbial community. After 50 days of silage fermentation, high-quality and low-quality samples were selected from every mixing ratio (90:10, 80:20, and 65:35). The silage chemical composition, lignocellulose degradation enzyme activity, microbial community composition, and potential functions were used to explore the relevance between silage quality and the characteristics of the microbial community. The contents of hemicellulose, neutral detergent fiber, pH, and the activities of endoglucanase and exoglucanase were significantly affected by mixing ratios and silage quality grade. There were higher crude protein content, lignocellulose degrading enzyme activity, and lower pH, lignin, and acid detergent fiber in the mixing of 65:35 (BP65%) samples. The PERMANOVA results showed that mixing ratios had significant impacts on microbial community composition and bacterial fermentation functions. There was a higher bacterial diversity, lower fungal diversity, and better functional potentials for fermentation and lignocellulose degradation in BP65% high-quality silage. The dominant genera were *Lactobacillus*, *Cladosporium*, and *Wallemia* in all samples. The relative abundance of *Clostridium*, *Rhodococcus*, *Turicibacter*, *Ralstonia*, and *Burkholderia* was significantly higher in BP65% high-quality samples. There was a higher abundance of *Wallemia* in the BP65% samples than in other mixing ratios samples. Notably, silage quality showed a close relationship with *Lactobacillus*, *Turicibacter*, *Romboutsia*, *Wallemia,* and *Pichia*. In summary, 65:35 was a suitable mixing ratio for *B. papyrifera* and wheat bran silage, but high-quality silage still required the participation of multiple specific rare microbial taxa. The higher bacterial diversity and specific microbial taxa abundance could be critical for improving *B. papyrifera* silage quality. We expect that our findings will provide new insights into silage quality driven by the characteristics of the microbial community.

## 1 Introduction

Paper mulberry (*Broussonetia papyrifera*) is an innovative woody feed known for its high yield and protein content, making it highly beneficial for ruminant animal feeding (Xiong et al., 2023). It is reported that the crude protein content accounts for 10–22% of the dry matter content in *B. papyrifera* (Zhang et al., 2019). In addition, due to its abundant medicinal bioactive substances, *B. papyrifera* is increasingly being used as a functional feed to promote animal growth and enhance immunity (Guo et al., 2021; Li et al., 2022). These characteristics of *B. papyrifera* make it suitable as a raw material for silage feed and help alleviate the feed supply crisis.

During natural silage fermentation, there are problems such as longer fermentation periods and unstable fermentation quality, but adding lactic acid bacteria (LAB) can solve these problems to some extent (Wang et al., 2023). LAB play an important role in the fermentation process of *B. papyrifera* silage feed and include various types of microbial taxa with high biodiversity (Sun et al., 2022). On the one hand, lactic acid production by LAB reduces the pH value of silage feed, inhibits the proliferation of spoilage microbes, and produces a sour aroma in silage feed, which is beneficial for livestock feeding (Sun and Song, 2020). On the other hand, LAB can also degrade lignocellulose in silage raw materials, increase the content of polysaccharides, and enrich the nutritional composition of feed (Guo et al., 2022). In practical production, more and more lactic acid bacteria are being used to prepare silage feed. Research has found that silage feed with added exogenous LAB often has better quality (Okoye et al., 2023). However, the formation of high silage feed quality required the participation of multiple microorganisms, not only relying on LAB but also other bacterial and fungal taxa (Ellis et al., 2016; Duniere et al., 2017). At the same time, the assembly process of microbial community is both deterministic and stochastic, which makes silage fermentation unstable. Therefore, the relationships between high-quality fermented silage and the characteristics of microbial communities are still unclear and require further research to clarify.

In addition, the high moisture content of the leaves can promote the growth of spoilage microorganisms when used directly in silage preparation, leading to unstable fermentation (Zhang et al., 2019; Guo et al., 2021). Mixing dry matter is one of the effective methods to reduce the moisture content of silage raw materials and improve the stability and success rate during silage fermentation (Wang et al., 2023; Guo et al., 2021). In actual production, wheat bran, due to its low price, and high content of dietary fiber, protein, various trace elements, and vitamins, is suitable for preparing silage feed in combination with *B. papyrifera* (Du et al., 2023). In addition to reducing the moisture content of silage materials, the mixture of *B. papyrifera* and wheat bran creates different ecological niches for various microbial communities, improving both diversity and fermentation function. Meanwhile, the quality of silage is significantly affected by the mixing ratio of raw materials (Du G. L. et al., 2021; Du Z. et al., 2021; Wang et al., 2022). Hence, it is critical to find a suitable mixing ratio to improve *B. papyrifera* silage quality.

Based on high-throughput sequencing technology of bacterial 16S and fungal ITS combined with silage quality analysis, lignocellulose degradation enzyme activity detection, microbial community structure, and fermentation functional potential were studied. The aim of this study is to explore *B. papyrifera* and wheat bran mixed silage quality driven by the characteristics of the microbial community and to develop the key microbial taxa that determine the silage quality. The research results will further enrich the microbial mechanisms that shape the quality of silage and provide a theoretical basis for the development of microbial agents for silage fermentation.

## 2 Materials and methods

### 2.1 Silage preparation

*B. papyrifera* was harvested in August 2021 from the 66-hectare planting base of Fengtang Ecological Agriculture Co., Ltd. (Taian, China). The silage materials were chopped into an approximate length of 1 cm with an automatic forage chopper and immediately taken to the laboratory. The fresh *B. papyrifera* (BP) and dry wheat bran (WB) were mixed at ratios of 90:10 (BP90%, WB10%), 80:20 (BP80%, WB20%), and 65:35 (BP65%, WB35%). A total of 500 g of fresh silage materials was mixed homogenously and packed into polyethylene bags (30 cm × 20 cm). Then, four types of *Lactobacillus* (*Lactobacillus plantarum*, *Lactobacillus rhamnosus*, *Lactobacillus paracasei*, *Lactobacillus casei*) were mixed in a 1:1:1:1 ratio, and 10% (w/v) of the inoculum was added to *B. papayrifera* (BP) and wheat bran different mixed proportions treatment groups. The concentration of each *Lactobacillus* was 1 × 10^8^ cfu/mL. Each type *Lactobacillus* was added with 12.5 mL and thoroughly mixed before being added to silage bags. Finally, the silage bags were vacuum-sealed and stored at room temperature (20–30°C) for 50 days of silage fermentation. After 50 days, high-quality and low-quality samples were selected from each mixing ratio based on the color, smell, and looseness of the feed (Abegunde et al., 2017). Five biological replicates were set for each sample group (Table 1). Five bags of each treatment were selected to determine the changes in silage chemical composition, lignocellulose degradation enzyme activity, microbial community composition, and potential functions.

**Table 1:** Categorization of silage fermentation quality.

Silage 50 d		BP90%H	BP90%L	BP80%H	BP80%L	BP65%H	BP65%L
Sensory evaluation	Color	Chartreuse	Turquoise	Chartreuse	Turquoise	Chartreuse	Turquoise
	Smell	Sour aroma	Mild sour	Sour aroma	Mild sour	Sour aroma	Mild sour
	Looseness	Loose and not touching hands	Slightly loose and not touching hands	Loose and not touching hands	Slightly loose and not touching hands	Loose and not touching hands	Slightly loose and not touching hands

BP90%H, the high-quality silage in BP and WB mixed at a ratio of 90:10; BP90%L, the low-quality silage in BP and WB mixed at a ratio of 90:10; BP80%H, the high-quality silage in BP and WB mixed at a ratio of 80:20; BP80%L, the low-quality silage in BP and WB mixed at a ratio of 80:20; BP65%H, the high-quality silage in BP and WB mixed at a ratio of 65:35; BP65%L, the low-quality silage in BP and WB mixed at a ratio of 65:35.

### 2.2 Silage chemical composition and enzyme activities analysis

The silage samples were dried at 65°C for at least 48 h until constant weight. Then, the dry samples were milled to pass through a 1.0-mm sieve for chemical composition analysis. The crude protein (CP) content was measured using the method of Kjeldahl (AOAC, 1990). The cellulose, hemicellulose, lignin, acid detergent fiber (ADF), and neutral detergent fiber (NDF) content in silage were determined using the method of Van Soest et al. (1991).

The activity of filter paper cellulase was determined using the dinitrosalicylic acid method (Colombatto et al., 2004). The activities of endoglucanase and exoglucanase were measured using the 3,5-dinitrosalicylic acid (DNS) method (Gupta et al., 2012). The activity of *β*-glucosidase was determined using the p-nitrophenol matrix method (Kourtev et al., 2002).

### 2.3 Microbial diversity analysis

The total microbial community genomic DNA extraction and quality test of each silage sample were performed according to the method of Wang et al. (2022). The V3–V4 region of the 16S ribosomal RNA (rRNA) gene was amplified with the polymerase chain reaction (PCR) thermocycler (ABI, CA, United States), with the primer 799F (AACMGGATTAGATACCCKG) and 1193R (ACGTCATCCCCAC CTTCC). The fungal primers ITS3/ITS4 were amplified with the primer pairs ITS3F (GCATCGATGAAGAACGCAGC) and ITS4R (TCCTCCGCTTATTGATATGC). The PCR programs were as follows: 95°C for 3 min; bacterial 27 and fungal 35 cycles of 95°C for 30 s, 55°C for 30 s, and 72°C for 45 s; 72°C for 10 min; and indefinite hold at 4°C upon completion. After DNA extraction, purification, and quality assessment, the PCR products were sent to Majorbio (Shanghai) for sequencing using the Illumina MiSeq-PE300 platform. All sample sequence quantities were unified by a minimum number before bioinformatics analysis. The bacterial and fungal taxonomy of each OUT representative sequence was analyzed using the 16S rRNA database (Silva v138) and ITS database (unite 8.0), based on a 0.7 threshold. The Chao and Shannon rarefaction curve was calculated by randomly resampling each sample several times, based on a 97% OTU sequence similarity threshold (Wang et al., 2024). After the rarefaction curve approached a plateau, they were used to evaluate the richness and diversity of the microbial community. In this study, FAPROTAX was used to predict the functional potential of bacterial metabolic or other ecological functions^1^ (Sansupa et al., 2021). BugBase was used to predict the functional potential of bacterial communities^2^ (Xiao et al., 2023). The raw sequencing data including all samples have been deposited into the NCBI Sequence Read Archive (SRA) database under the Accession Number of PRJNA1119582.

### 2.4 Statistical analysis

The statistical analyses in our study were performed by SPSS software (SPSS Inc., Chicago, IL). Two-way analysis of variance (two-way ANOVA) was used to check the significant differences in silage quality, enzyme activity, and microbial community diversity among different mixing ratios of *B. papyrifera* and wheat bran. The diversity index of Chao and Shannon was performed by Mothur software (Li et al., 2022). The Kruskal–Wallis H-test was used to determine the differences in Chao, Shannon, and bacterial community potential fermentation functions among different samples (MacFarland and Yates, 2016). Principal coordinates analysis (PCoA) was performed using the R “vegan” package based on Bray–Curtis dissimilarity distances to show the differences in microbial community composition among the samples (Wang et al., 2024). Spearman’s correlation heatmaps among microbial taxa, silage chemical composition, and enzyme activities were performed using R software (Miller and Bishop, 2021).

## 3 Results

### 3.1 Silage chemical composition and enzyme activity

The characteristics of silage feed after 50 days of ensiling are shown in Table 2. The two-way ANOVA analysis results indicated that the contents of hemicellulose, neutral detergent fiber, and pH were significantly affected by different mixing ratios and quality grades (*p* < 0.05). All silage chemical composition contents were significantly affected by the mixing ratios (*p* < 0.05). There were higher crude protein content and lower pH, lignin, and acid detergent fiber in the mixing of 65:35 (BP65%) samples. In addition, there were higher hemicellulose, neutral detergent fiber, and lower lignin content in the high-quality samples with different mixing ratios.

**Table 2:** Chemical property after 50 days of silage.

	Crude protein (g/100 g)	Cellulose (g/100 g)	Hemicellulose (g/100 g)	Lignin (g/100 g)	Acid detergent fiber (g/100 g)	Neutral detergent fiber (g/100 g)	pH
BP90%H	5.37 ± 0.12c	8.20 ± 0.17a	7.20 ± 0.17a	1.93 ± 0.06bc	10.17 ± 0.12a	17.37 ± 0.25a	4.68 ± 0.04d
BP90%L	4.92 ± 0.14d	6.60 ± 0.26b	6.23 ± 0.15b	2.17 ± 0.15b	8.90 ± 0.26b	15.13 ± 0.15b	4.94 ± 0.02a
BP80%H	5.84 ± 0.17b	6.80 ± 0.30b	5.23 ± 0.12c	1.93 ± 0.15bc	8.77 ± 0.31b	14.10 ± 0.20c	4.88 ± 0.02b
BP80%L	5.66 ± 0.13b	4.63 ± 0.12d	3.63 ± 0.25e	3.87 ± 0.21a	8.67 ± 0.15b	12.27 ± 0.15e	4.77 ± 0.04c
BP65%H	7.85 ± 0.09a	5.53 ± 0.15c	6.37 ± 0.21b	1.27 ± 0.15d	7.03 ± 0.25d	13.40 ± 0.26d	4.04 ± 0.03f
BP65%L	8.05 ± 0.04a	5.67 ± 0.06c	4.17 ± 0.15d	1.77 ± 0.15c	7.57 ± 0.15c	11.73 ± 0.29f	4.42 ± 0.03e

Values were means ± SD. Values in the same column with different letters are significantly different.

The enzyme activities of silage feed after 50 days of ensiling are shown in Table 3. The two-way ANOVA analysis results showed that the activities of endoglucanase, and exoglucanase were significantly affected by different mixing ratios and quality grades (*p* < 0.01). All lignocellulose degrading enzyme activities were significantly affected by the mixing ratios (*p* < 0.05). There were higher lignocellulose activities in the mixing of 65:35 (BP65%) samples, especially in BP65%L samples. As the proportion of wheat bran increased, the activity of exoglucanase significantly improved.

**Table 3:** Activities of lignocellulose degradation enzymes.

	Filter paper cellulase (U/mL)	endoglucanase (U/mL)	exoglucanase (U/mL)	β-glucosidase (U/mL)
BP90%H	10.37 ± 0.50d	22.91 ± 0.56 cd	3.20 ± 0.57e	19.96 ± 0.25c
BP90%L	10.22 ± 0.40d	18.98 ± 0.55f	0.55 ± 0.18f	19.51 ± 0.45c
BP80%H	11.53 ± 0.18c	23.64 ± 0.56c	6.60 ± 0.14d	22.81 ± 0.49b
BP80%L	10.45 ± 0.51d	22.04 ± 0.52d	7.57 ± 0.22c	22.66 ± 0.99b
BP65%H	172.26 ± 0.60b	484.85 ± 0.45b	40.10 ± 0.26b	22.15 ± 0.61b
BP65%L	196.56 ± 0.53a	545.18 ± 0.69a	53.13 ± 0.30a	26.39 ± 0.74a

Values were means ± SD. Values in the same column with different letters are significantly different.

### 3.2 Microbial community diversity and composition

**Figure 1:**
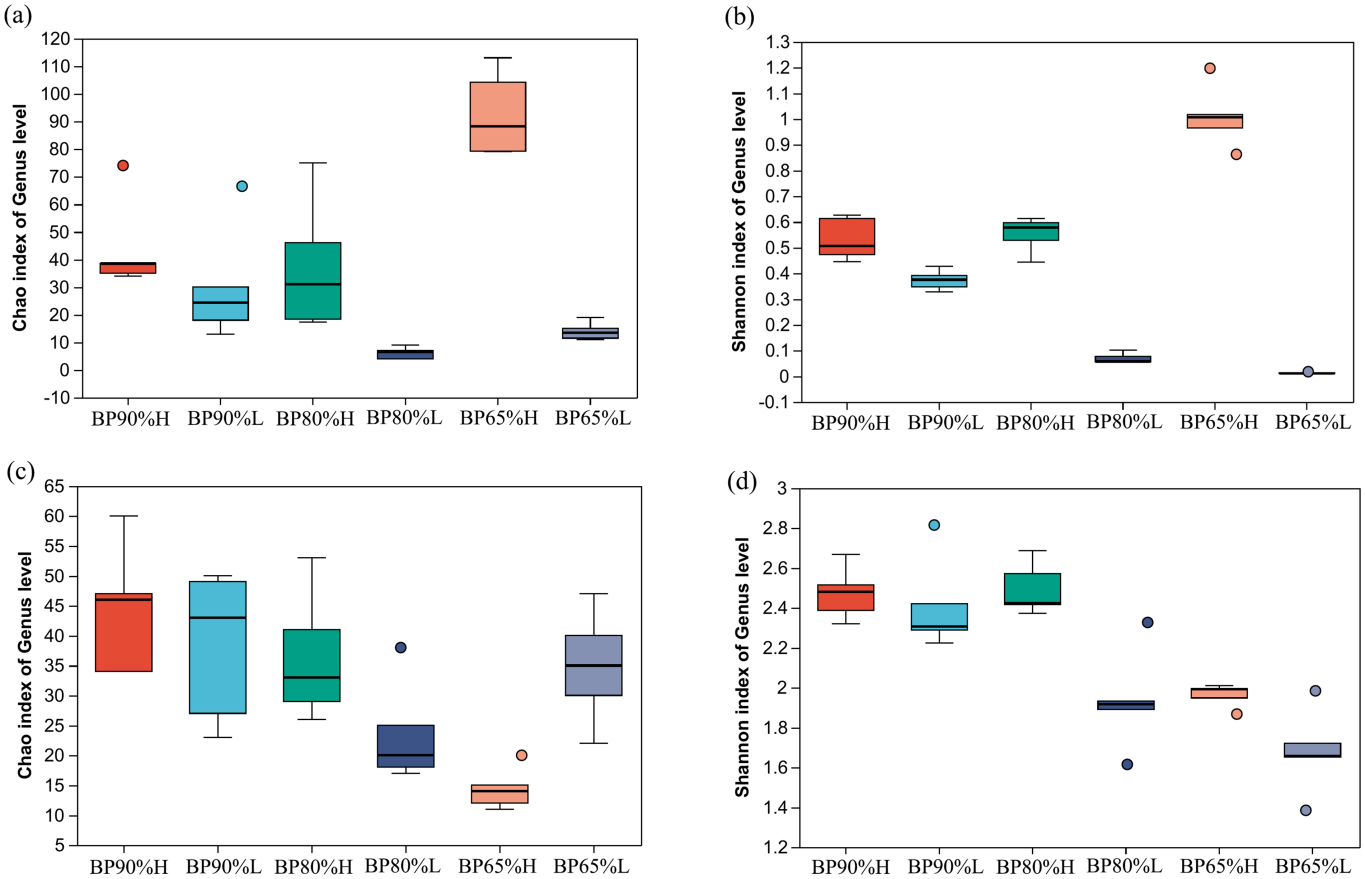
Chao and Shannon indices of bacterial and fungal communities. **(A)** Chao index of bacterial community; **(B)** Shannon index of bacterial community; **(C)** Chao index of fungal community; and **(D)** Shannon index of fungal community.

Based on the analysis of inter-group differences in the Chao and Shannon index of bacterial community, it was found that the Chao and Shannon indices in BP65%H samples were significantly higher than other samples (*p* < 0.01) (Figures 1A,B), while the Chao and Shannon indices were lowest in BP80%L samples. For the fungal community, the Chao indices were significantly lower in BP65%H than in other samples (*p* < 0.01) (Figure 1C). The Shannon indices were significantly lower in BP65%H versus BP90%H, BP90%L, and BP80%H (*p* < 0.05) (Figure 1D). These results indicated that there was higher bacterial community richness and lower fungal community richness in BP65%H. For community alpha diversity, bacterial diversity was higher, while fungal was lower in BP65%H.

**Figure 2:**
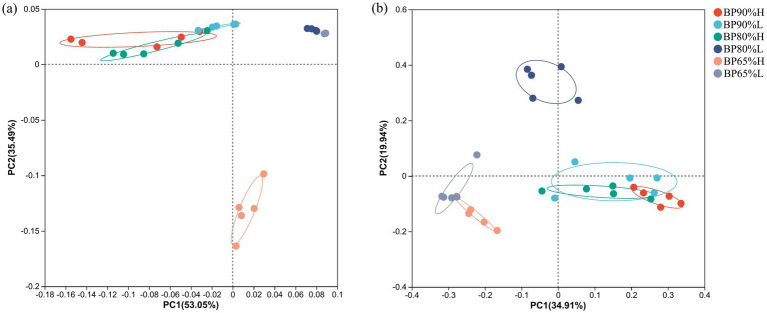
Principal coordinates analysis (PCoA) of bacterial **(A)** and fungal **(B)** community compositions.

The PCoA and ANOSIM results indicated that there were significant differences in bacterial community composition between BP65% and other mixing radios samples (*R* = 0.79, *p* = 0.001) (Figure 2A). In particular, the bacterial community composition in BP65%H samples was clearly distinguished from other samples. Meanwhile, there were similar compositions between BP80%L and BP65%L samples.

For the fungal community, there were also significant differences between BP65% and other mixing radios samples (*R* = 0.85, *p* = 0.001) (Figure 2B). The fungal community composition in BP80%L samples was distinguished from other samples. Different from the composition of the bacterial community, there were similar compositions between BP65%H and BP65%L samples.

**Figure 3:**
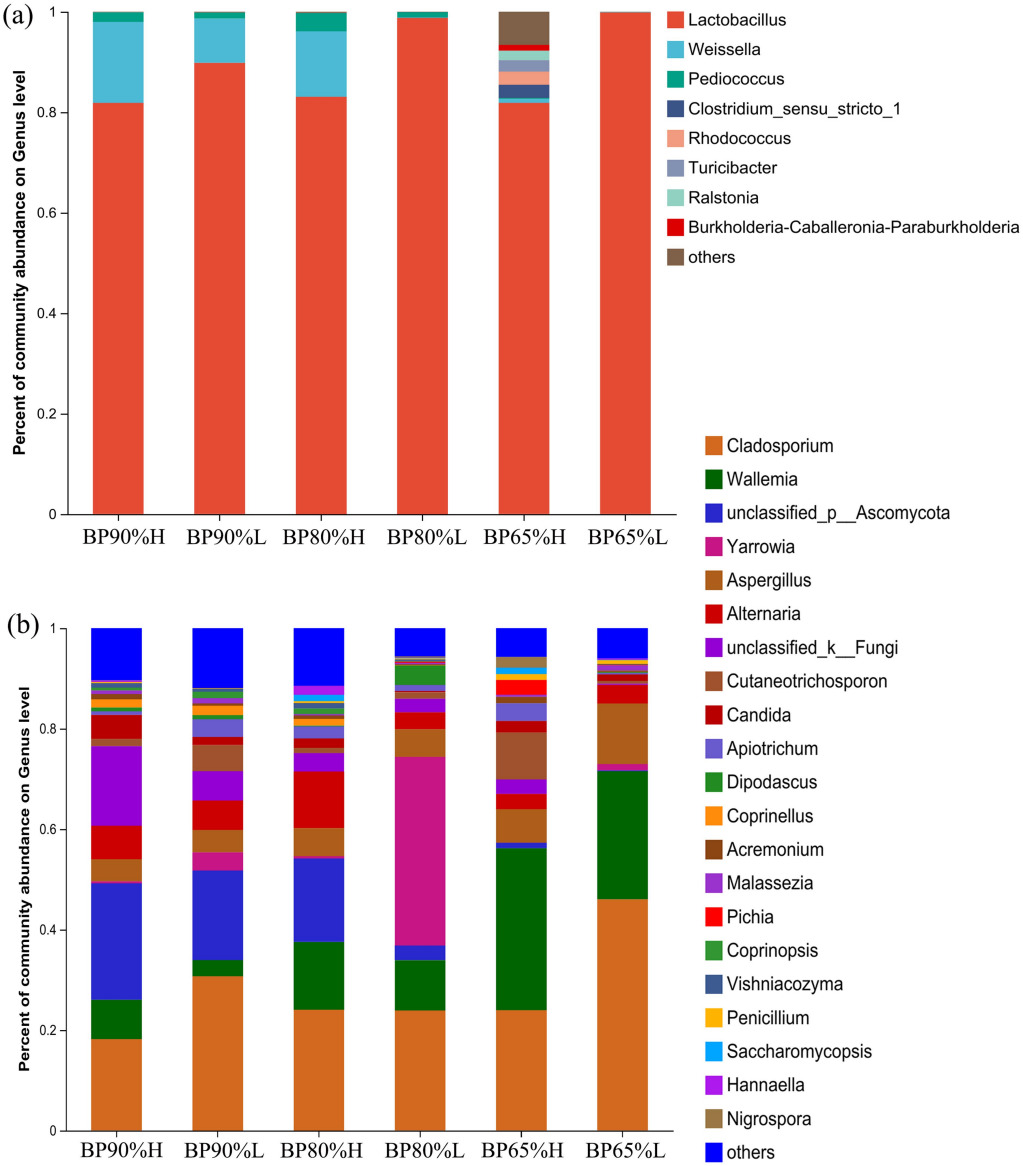
Bacterial **(A)** and fungal **(B)** community structures at the level of genus.

As shown in Figure 3A, *Lactobacillus* (89.21%) was the dominant genus in all samples, followed by *Weissella* (6.46%) and *Pediococcus* (1.34%). It was noteworthy that the abundance of *Clostridium*, *Rhodococcus*, *Turicibacter*, *Ralstonia*, and *Burkholderia* was significantly higher in BP65%H samples than in the other samples. These results were consistent with the higher diversity in BP65%H samples. The PERMANOVA results showed that there were significant differences in the bacterial community composition among different samples (R2 = 0.89, *p* = 0.001). For the fungal community, *Cladosporium* (27.80%), *Wallemia* (15.39%), and *Aspergillus* (6.45%) were the dominant genus in all samples (Figure 3B). The PERMANOVA results showed that different mixing ratios had significant impacts on the composition of the fungal community (*R*^2^ = 0.69, *p* = 0.001). The relative abundance of *Wallemia* was higher in all high-quality samples versus low-quality samples. There was a higher abundance of *Wallemia* in the BP65% samples than in other mixing ratios samples (*p* < 0.05).

### 3.3 Bacterial community potential fermentation functions

**Figure 4:**
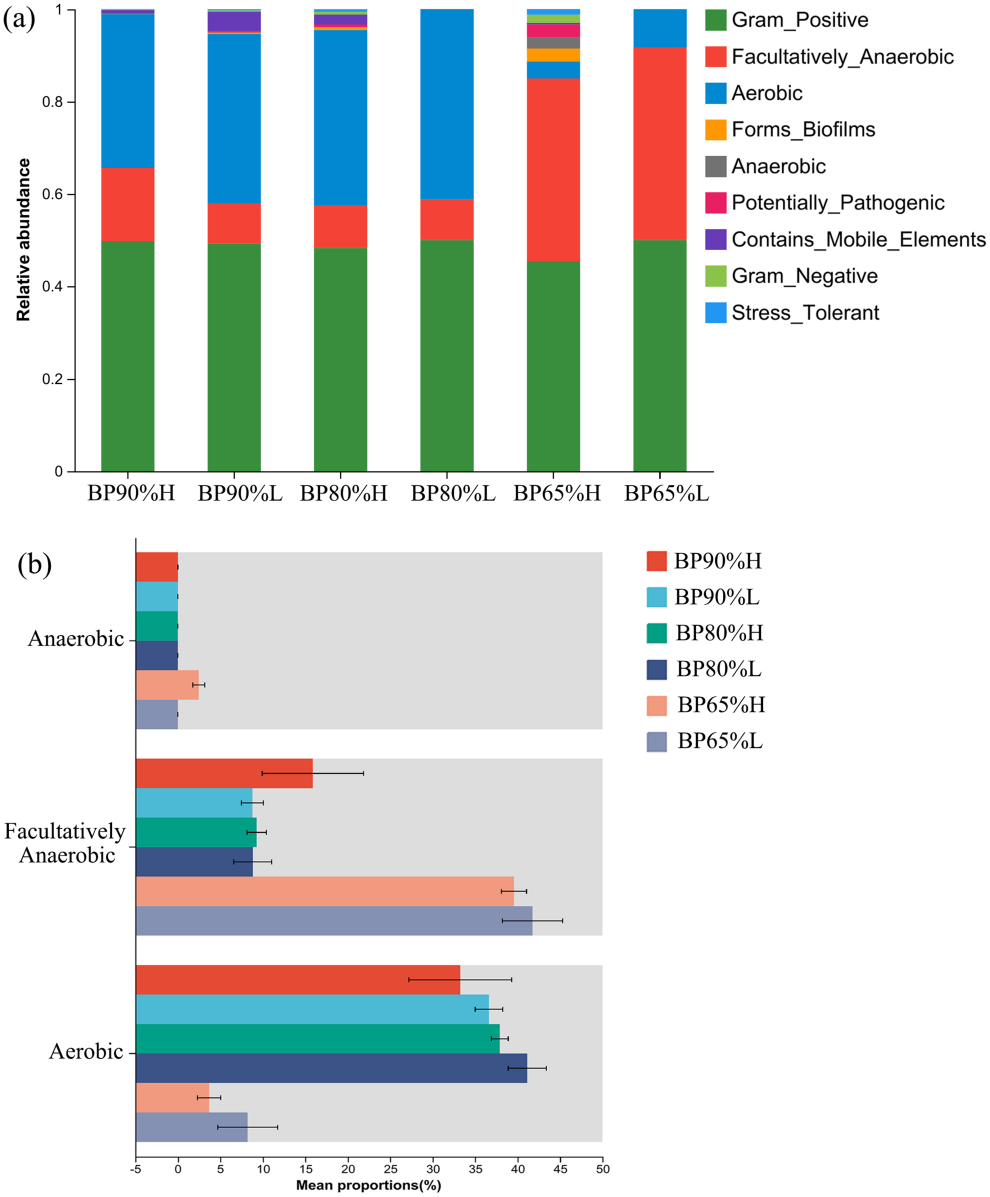
Variations in bacterial community composition of phenotype. **(A)** Functional prediction of bacterial community FAPROTAX and **(B)** test for inter-group differences in predictive function.

Based on BugBase phenotype prediction analysis, there were significant variations in bacterial community composition of phenotype among different samples (Figure 4A). The relative abundances of facultatively anaerobic were significantly higher in BP65% samples than in other samples (*p* < 0.01), while the proportions of aerobic were opposite in BP65%. Interestingly, we found a rich anaerobic function in the BP65%H sample. The Kruskal–Wallis H-test results showed that the proportions of anaerobic function were significantly higher in BP65%H versus other samples (*p* < 0.05) (Figure 4B). The functions of aerobic were significantly lower in BP65% mixing radio than others (*p* < 0.01), while the functions of facultatively anaerobic were opposite in BP65% (*p* < 0.01). These indicated that the bacterial community in BP65% was mainly composed of anaerobic and facultatively anaerobic fermentation functional groups.

**Figure 5:**
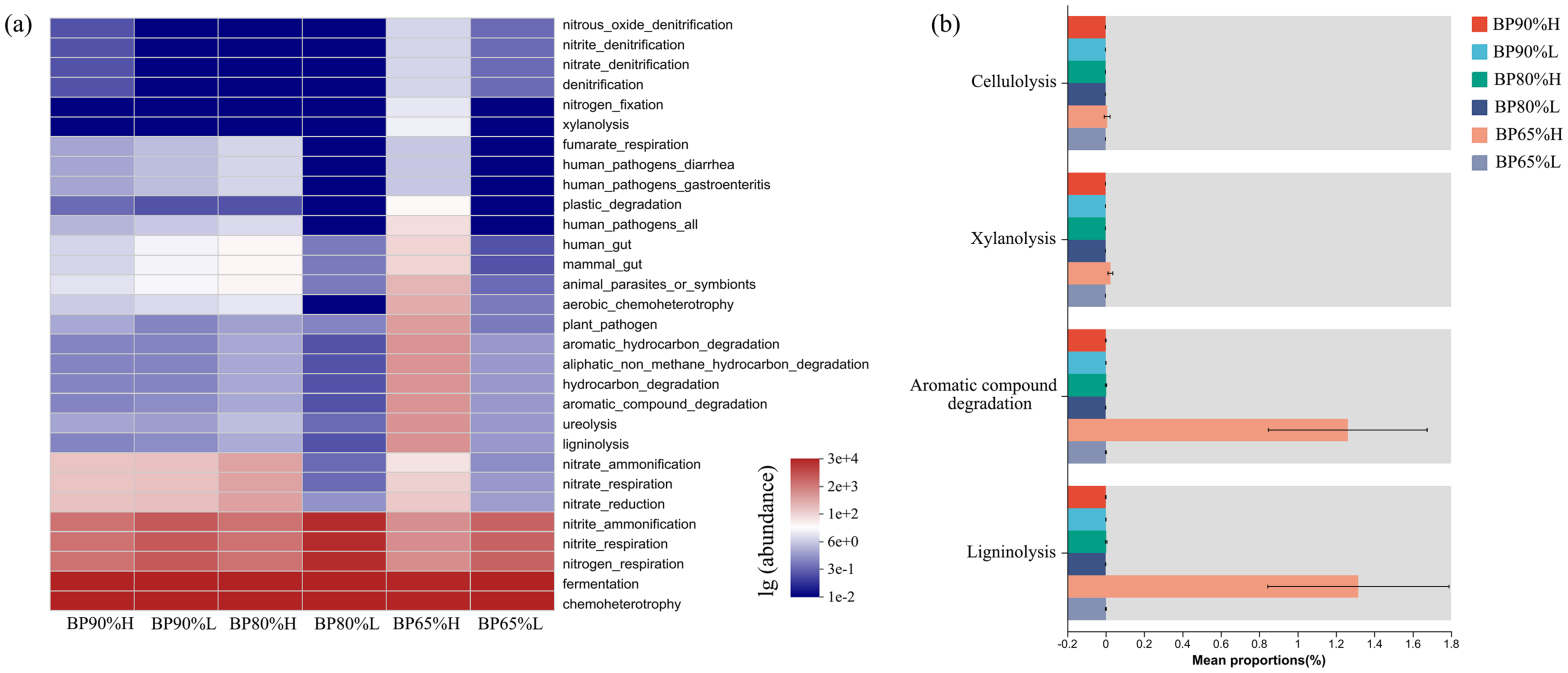
Bacterial community FAPROTAX functions prediction **(A)** and functional group difference analysis **(B)**.

The FAPROTAX functions prediction results showed that bacterial community functions were mainly composed of fermentation and chemoheterotrophy (Figure 5A). It was worth noting that the average abundance of bacteria involved in ligninolysis, ureolysis, aromatic compound degradation, and hydrocarbon degradation was higher in BP65%H than in other samples. The Kruskal–Wallis H-test results showed that the proportions of bacterial cellulolysis, xylanolysis, aromatic compound degradation, and ligninolysis functions were significantly higher in BP65%H (*p* < 0.05) (Figure 5B). This indicated that there were better functional potentials for the lignocellulose degradation in BP65%H.

### 3.4 Relationship between microbial taxa and silage quality

**Figure 6:**
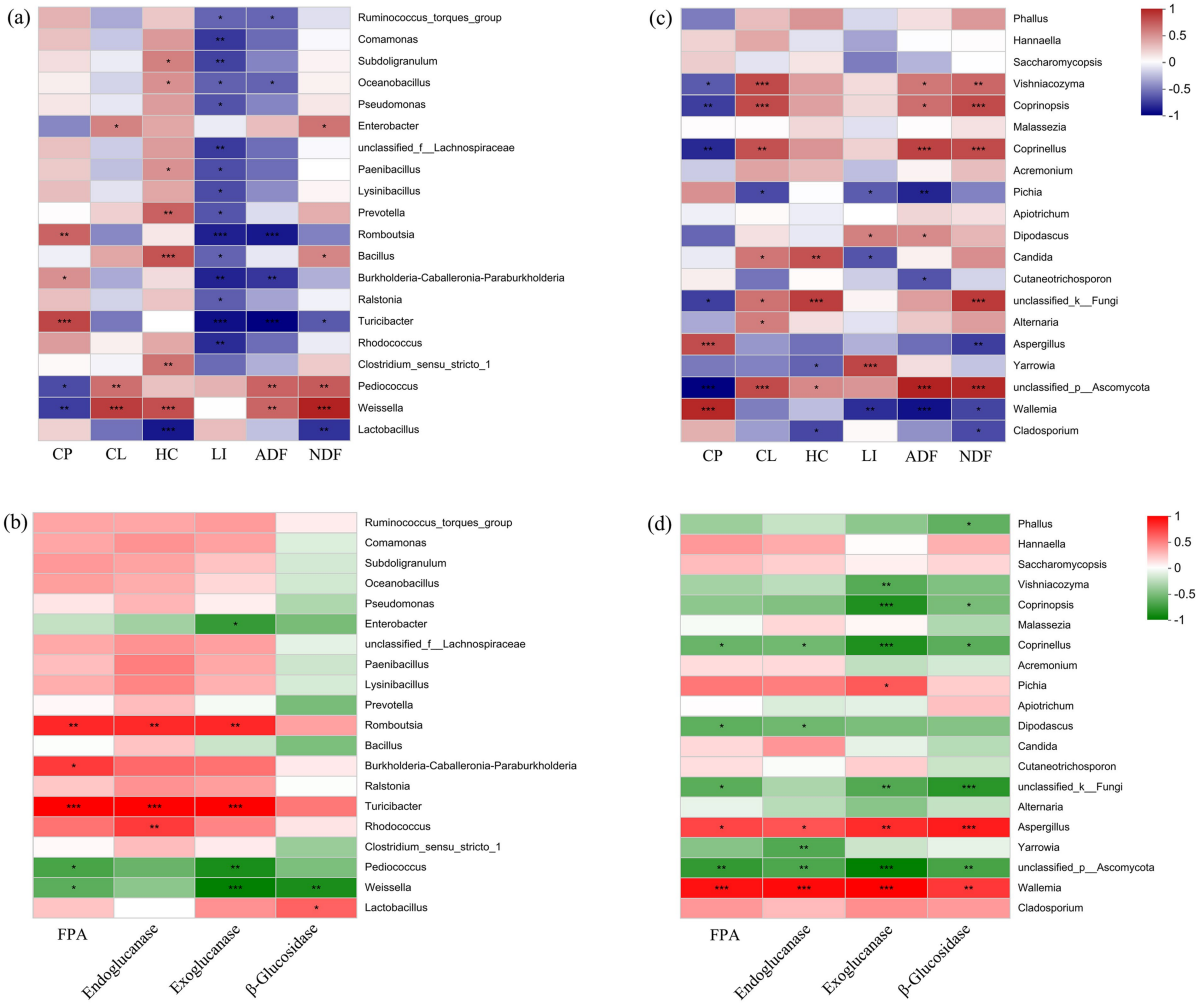
Correlation heatmap of microbial taxa and silage quality properties. **(A)** Bacterial community and lignocellulose content; **(B)** bacterial community and lignocellulose degradation enzymes; **(C)** fungal community and lignocellulose content; and **(D)** fungal community and lignocellulose degradation enzymes. FPA, filter paper cellulase CP, crude protein; CL, cellulose; HC, hemicellulose; LI, lignin; ADF, acid detergent fiber; NDF, neutral detergent fiber. *p < 0.05, **p < 0.01, ***p < 0.001.

The correlation heatmap revealed significant negative correlations between the abundance of *Lactobacillus* and hemicellulose (HC), as well as acid detergent fiber (ADF) content (*p* < 0.01). In contrast, the abundance of *Weissella* and *Pediococcus* was significantly positively correlated with crude protein (CL), HC, acid detergent fiber (ADF), and NDF content (*p* < 0.05) (Figure 6A). In terms of enzyme activity, there were significant positive correlations between the abundance of *Lactobacillus* and *β*-glucosidase activity (*p* < 0.05) (Figure 6B). Except for *Lactobacillus*, the abundance of *Turicibacter* and *Romboutsia* was significantly positively correlated with the activity of filter paper cellulase (FPA), endoglucanase, and exoglucanase (*p* < 0.01). On the contrary, the abundance of *Weissella* and *Pediococcus* was significantly negatively correlated with the activities of exoglucanase and β-glucosidase (*p* < 0.05). For the fungal community, the abundance of dominant taxa *Cladosporium*, *Wallemia*, *Aspergillus,* and *Pichia* was significantly negatively correlated with the content of lignocellulose components (Figure 6C). In terms of enzyme activity correlation, there were significant positive correlations between the abundance of *Wallemia*, *Aspergillus*, *Pichia,* and the activity of FPA, endoglucanase, exoglucanase, and β-glucosidase (*p* < 0.05) (Figure 6D).

## 4 Discussion

### 4.1 The effects of *Broussonetia papyrifera* and wheat bran mixing ratios on the silage quality and the characteristics of the microbial community

Research has found that the quality of silage is significantly affected by the mixing ratio of raw materials (Liu et al., 2023). In general, the quality of silage was related to pH value, lactic acid, lignocellulose, and crude protein content. The high-quality silage often contains lower pH and lignocellulose content, as well as higher lactic acid and crude protein content (Ni et al., 2014). Our study also found that the contents of hemicellulose, neutral detergent fiber, and pH were significantly affected by quality grade. The contents of crude protein, cellulose, hemicellulose, lignin, acid detergent fiber, neutral detergent fiber, and pH were significantly affected by *B. papyrifera* and wheat bran’s different mixing ratios. There were higher crude protein content and lower pH, lignin, and acid detergent fiber in the mixing of 65:35 (BP65%) samples. This may be due to the higher lignocellulose activity in the mixing of 65:35 (BP65%) samples. In addition, there were higher hemicellulose, neutral detergent fiber, and lower lignin content and pH in the high-quality samples with different mixing ratios. On the one hand, mixed silage directly adjusts the chemical composition of the raw materials to improve the silage quality. On the other hand, it indirectly alters the silage quality by affecting the composition and function of the microbial community (Liu et al., 2023; Ni et al., 2017).

It is generally believed that mixed silage alters the alpha diversity of bacterial communities by providing abundant nutrients and creating more ecological niches for microorganisms (Xin et al., 2022). Moreover, the mixing ratios between different silage materials could significantly affect the microbial community composition, diversity, and abundance of specific microbial groups during the silage fermentation process (Chi et al., 2022). The PERMANOVA results showed that different mixing ratios had significant impacts on the composition of bacterial and fungal communities. However, the assembly process of the microbial community is both deterministic and stochastic; the abundant bacterial taxa were more strongly determined using deterministic processes, while rare bacterial taxa were more strongly determined using stochastic processes (Stegen et al., 2012; Liu et al., 2021). Hence in our silage samples with the same mixing ratio, there were significantly different microbial community structures. The Chao and Shannon indices of the bacterial community in BP65%H samples were significantly higher than in other samples. Some potential beneficial bacterial taxa, *Turicibacter* and *Ralstonia* were only found in BP65%H samples. This indicated that the formation of high-quality silage may require the joint participation of multiple specific groups. Meanwhile, the previous study has also found that adding exogenous LAB or mixed silage can reduce bacterial alpha diversity in silage fermentation, which may be due to the addition of a predominant genus of *Lactobacillus* (Xin et al., 2022; Ogunade et al., 2018). This finding was consistent with our study that *Lactobacillus* was the dominant genus in all samples and played important roles in silage fermentation. For the fungal community, the Chao and Shannon indices were lower in BP65%H than in other samples. This may be related to the higher bacterial alpha diversity and lower pH in the BP65% H sample.

Silage fermentation is primarily dominated by bacterial communities, and predicting their functional characteristics helps evaluate the impact of microbes on silage quality (Wang et al., 2020). Our research found that the mixing ratios not only significantly altered the composition of the microbial community but also significantly affected the fermentation potential of the bacterial community. The relative abundances of facultatively anaerobic were significantly higher in 65%BP samples than in other samples, while the proportions of aerobic were the opposite. Many microbial taxa identified in silages are facultative anaerobes, and they can produce lactic acid or degrade lignocellulose during the fermentation process to improve the quality of silage (Kim et al., 2021). While aerobic fermentation is mainly carried out by aerobic bacteria (AB), yeast, and mold, whose growth and reproduction can lead to a decrease in the quality of silage (Okoye et al., 2023). These results were related to specific microbial community composition in different samples. It was worth noting that there were better functional potentials for fermentation and lignocellulose degradation in 65%BPH. This may be due to the presence of suitable microbial community composition and key microbial taxa for silage in 65% BPH samples. Our study indicated that 65:35 was a suitable mixing ratio for *B. papyrifera* and wheat bran silage, but high-quality silage still required the participation of multiple specific rare microbial taxa.

### 4.2 Exploring the relationship between the key microbial taxa and *Broussonetia papyrifera*/wheat bran mixed silage quality

Research has found that the quality of silage feed is closely related to microbial community composition, especially the types and abundance of lactic acid bacteria (LAB) (Sun et al., 2012). LAB include various types of microbial taxa with high biodiversity (Liu et al., 2014). It is reported that *Lactobacillus*, *Enterococcus*, *Pediococcus*, *Weissella,* and *Lactococcus* were beneficial to improve silage quality (Wang et al., 2022; Driehuis et al., 2018). LAB play an important role in producing lactic acid and reducing pH, while *Weissella* has a strong inhibitory effect on harmful microbes (Sun et al., 2012; Ndagano et al., 2011). Our study found that *Lactobacillus* was the dominant genus in all samples, followed by *Weissella* and *Pediococcus* after exogenous addition. However, there were differences in the abundance of *Lactobacillus*, *Weissella*, and *Pediococcus* among different mixing ratios, indicating that the assembly process of microbial community was influenced by both exogenous addition and mixing ratios (Sun et al., 2022; Li et al., 2024). In addition to the common beneficial microbial groups for silage fermentation, our research also identified microbial taxa with the potential to improve silage quality. The correlation analysis results showed that there were significant negative correlations between the abundance of *Lactobacillus*, *Turicibacter*, *Romboutsia,* and lignocellulose component content. In terms of enzyme activity, the abundance of *Lactobacillus*, *Turicibacter,* and *Romboutsia* was also significantly positively correlated with lignocellulose degrading enzyme activity. The genus *Turicibacter* was considered a lactic acid-producing bacterium that could ferment lignocellulose to increase the carbohydrate content in feed (Lyu et al., 2022; Castillo-Lopez et al., 2020). The genus *Romboutsia* was reported to be engaged in the fermentation of carbohydrates under anaerobic respiration (Gerritsen, 2015). In general, most LAB are not able to degrade lignocellulose; however, *Turicibacter* and *Romboutsia* can provide more carbohydrates to support the reproduction of LAB (Axelsson, 2004). Hence, the synergistic effect of *Lactobacillus*, *Turicibacter,* and *Romboutsia* may play important roles in improving silage quality.

Fungi are generally believed to have adverse effects on the quality of silage, such as *Cladosporium*, and *Aspergillus*, while some fungal taxa have also been found to be beneficial for silage fermentation (Ávila and Carvalho, 2019). Our study found that the abundance of dominant taxa *Wallemia* and *Pichia* was significantly negatively correlated with the content of lignocellulose but positively correlated with FPA, endoglucanase, exoglucanase, and *β*-glucosidase. It was reported that the genus *Pichia* could secrete hydrolytic enzymes to improve lignocellulose degradation rate and silage quality (Ren et al., 2024). In addition, the relative abundance of *Wallemia* was higher in high-quality samples versus low-quality samples. Research has found that *Wallemia* can promote acetic acid fermentation and inhibit the growth and reproduction of undesirable fungi during the silage fermentation process (Geiser et al., 2006; Du G. L. et al., 2021; Du Z. et al., 2021). Hence, *Wallemia* and *Pichia* may improve silage quality by inhibiting mold growth or decomposing lignocellulose to produce abundant carbohydrates. In our study, while *Wallemia* and *Pichia* can be beneficial, certain species may also lead to spoilage if silage raw materials or conditions change, potentially leading to the production of undesirable compounds.

The formation of high-quality silage requires the participation of multiple microorganisms, and the addition of exogenous strains can significantly affect the microbial diversity during the silage process (Duniere et al., 2017). The microbial diversity in silage fermentation can determine the silage quality (Ridwan et al., 2015). The higher bacterial alpha diversity contributes to the accumulation of metabolites, while higher fungal alpha diversity often leads to the accumulation of fungal toxins, reducing the stability and quality of silage (Wang et al., 2021). Our study found that the bacterial Chao and Shannon indices in BP65%H samples were significantly higher than in other samples. However, the fungal Chao indices were significantly lower in BP65%H than in other samples. These indicated that a higher diversity of bacterial community and a lower diversity of fungal community were prerequisites for preparing high-quality silage feed.

## 5 Conclusion

The quality of silage feed depends on both the raw materials used and the microbial community involved in the fermentation process. Therefore, the chemical composition of silage materials and the characteristics of the microbial community can affect the fermentation quality. Our study found that *B. papyrifera* and wheat bran mixing ratios had significant impacts on silage quality and the characteristics of the microbial community. The contents of hemicellulose, neutral detergent fiber, pH, and the activities of endoglucanase and exoglucanase were significantly affected by mixing ratios and silage quality grade. There were higher crude protein content and lower pH, lignin, and acid detergent fiber in the mixing of 65:35 (BP65%) samples. There was a higher bacterial diversity, lower fungal diversity, and better functional potentials for fermentation and lignocellulose degradation in BP65% high-quality silage. The formation of high-quality silage is closely related to *Lactobacillus*, *Turicibacter*, *Romboutsia*, *Wallemia*, and *Pichia*. In summary, the appropriate mixing ratio, higher bacterial diversity, and specific microbial taxa could be critical for improving *B. papyrifera* silage quality.

## Data Availability

The datasets presented in this study can be found in online repositories. The names of the repository/repositories and accession number(s) can be found in the article/Supplementary material.

## Footnotes

^1^ http://www.loucalab.com/archive/FAPROTAX/

^2^ https://bugbase.cs.umn.edu/index.html
