# Supplementary material for: Formation of high-quality mixed silage from paper mulberry and wheat bran driven by the characteristics of the microbial community
Source: Front Microbiol. 2024 Dec 13;15:1476067. doi: 10.3389/fmicb.2024.1476067 (PMC11671512; doi:10.3389/fmicb.2024.1476067)
Supplement: Supplementary file 1 [file Table_1.DOCX]

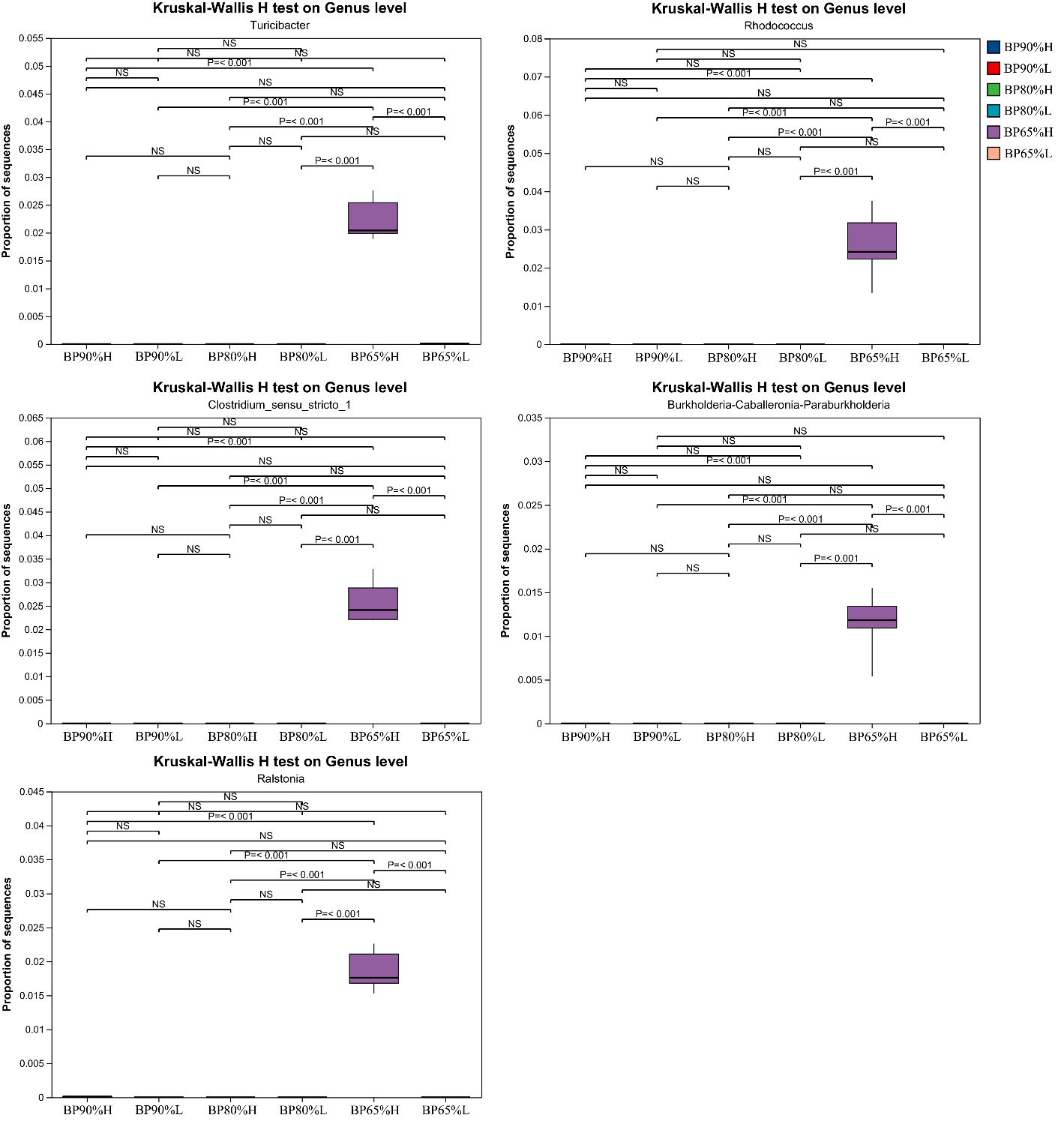


Fig. S1 The significance test results for differences of *Clostridium*, *Rhodococcus*, *Turicibacter*, *Ralstonia*, and *Burkholderia*.


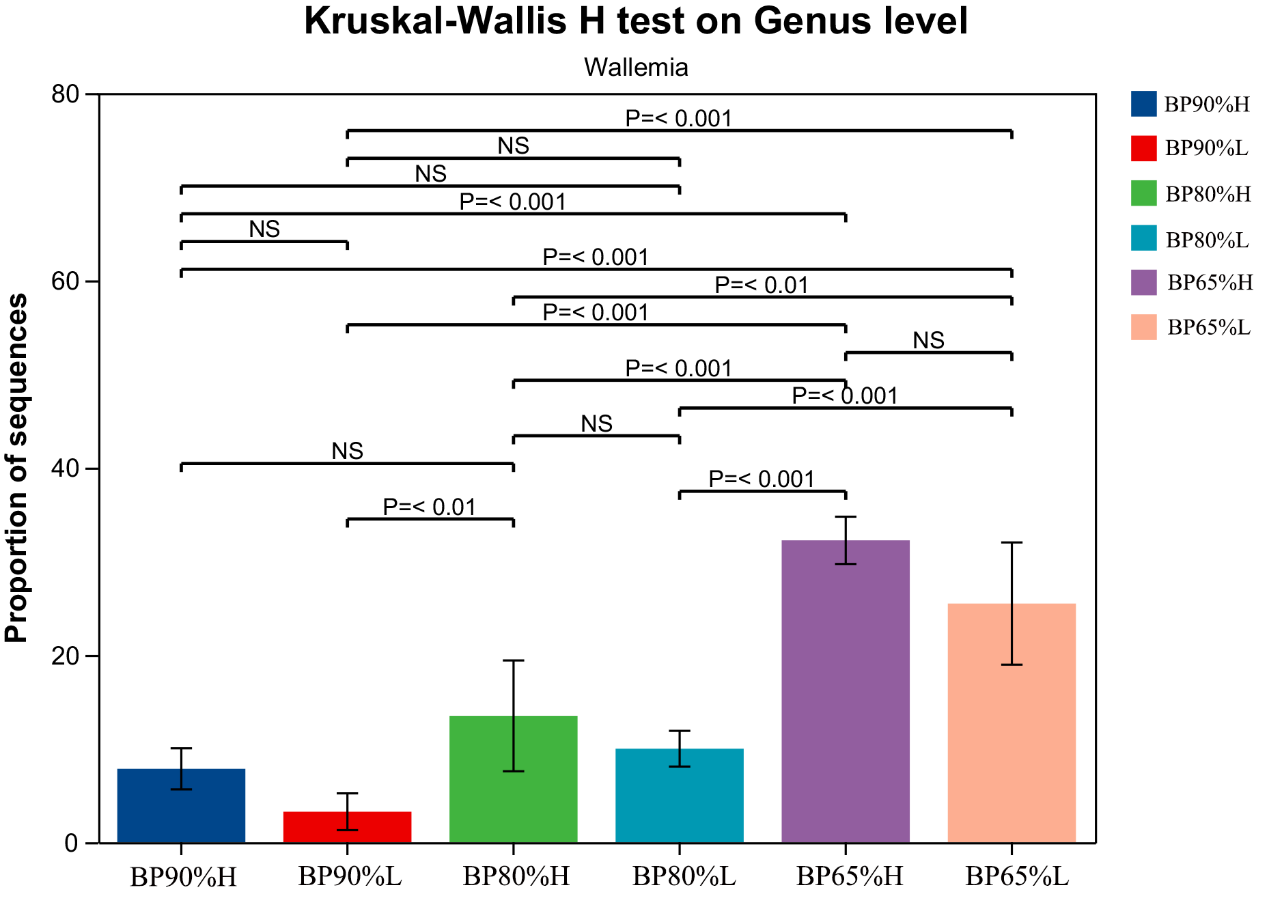


Fig. S2 The significance test results for differences of *Wallemia*.
